# New heights in CT differentiation of adrenal lesions and a rational definition of non-enhancement

**DOI:** 10.1186/s12880-025-01916-6

**Published:** 2025-09-26

**Authors:** Lichun Liu, Fangmei Zhu, Zongfeng Niu, Zongyu Xie, Dengfa Yang, Jian Wang, Cheng Yan

**Affiliations:** 1 Department of Electrocardiograph, Tongde Hospital of Zhejiang Province Affiliated to Zhejiang Chinese Medical University (Tongde Hospital of Zhejiang Province), Hangzhou, Zhejiang China; 2 Department of Radiology, Affiliated Hangzhou First People’s Hospital, Westlake University School of Medicine, Hangzhou, Zhejiang China; 3 Department of Radiology, Sir Run Run Shaw Hospital, Zhejiang University School of Medicine, Hangzhou, Zhejiang China; 4 Department of Radiology, The First Affiliated Hospital of Bengbu Medical University, Bengbu Anhui, China; 5 Department of Radiology, Taizhou Municipal Hospital, Taizhou, Zhejiang China; 6 Department of Radiology, Tongde Hospital of Zhejiang Province Affiliated to Zhejiang Chinese Medical University (Tongde Hospital of Zhejiang Province), No.234, Gucui Road, Hangzhou, Zhejiang PR China

**Keywords:** Adrenal, Adenoma, Cyst, Adrenal ganglioneuroma, Computed tomography

## Abstract

**Background:**

To explore the stratification and identification of adrenal lipid-poor adenomas (LPAs), adrenal cysts (ACs), and adrenal ganglioneuromas (AGNs) from each other *using contrast-enhanced* computed tomography (*CT)*.

**Methods:**

Pathologically confirmed, 348 patients were categorized into Model 1 (260 LPAs, 34 ACs), Model 2 (260 LPAs, 54 AGNs), and Model 3 (34 ACs, 54 AGNs). Statistical analyses were performed on the differences in the degree of enhancement in the arterial/venous phase (DEap/DEvp) (in HU) and the corresponding graded variables for the arterial/venous phase (GVap/GVvp). Models were evaluated via receiver operating characteristic (ROC) curves, calibration curves, and the Hosmer‒Lemeshow (HL) test.

**Results:**

The values of the area under the curve (AUC) for DEap, DEvp, GVap, and GVvp in Models 1–3 were 0.996, 1.000, 0.993, and 0.999; 0.980, 0.978, 0.961, and 0.975; and 0.734, 0.892, 0.725, and 0.883, respectively. The p values of the HL test were 0.984, 1.000, and 0.113, respectively. The DEvp interval values (in HU) for the LPAs, ACs, and AGNs were [4.9, 190.2] HU, [-3.7, 4.2] HU, and [-4.8, 41.8] HU, respectively. The GVap and GVvp ranges for the LPAs, ACs, and AGNs were [1, 6], [0, 2], and [0, 2] and [1, 6], [0, 1], and [0, 5], respectively.

**Conclusions:**

DEvp enhanced discrimination in Models 1 and 3, whereas DEap performed better in Model 2. Lesions with DEvp < 4.5 HU are likely represent non-enhancing pathology (e.g., cysts). When both GVap and GVvp are 0, when both GVap and GVvp are [2, 6], and when GVap is [3, 6] and GVvp is 6, LPA, AC, and AGN are excluded.

**Clinical trial number:**

Not applicable.

**Supplementary Information:**

The online version contains supplementary material available at 10.1186/s12880-025-01916-6.

## Introduction

The rising utilisation of modern medical imaging has increased detection of adrenal incidentalomas. These lesions—typically identified incidentally during unrelated examinations—demonstrate a 3–8% prevalence on computed tomography (CT), with adrenal adenomas representing approximately 30% of cases [1, 2]. Lipid-poor adenomas (LPAs) pose particular diagnostic challenges, whereas adrenal cysts (ACs) are exceptionally rare (incidence ∼0.06%) [3]. Similarly uncommon are adrenal ganglioneuromas (AGNs), benign extrarenal medullary tumours accounting for 0.3–2% of adrenal masses [4].

Endocrine guidelines endorse the 10 Hounsfield unit (HU) threshold on unenhanced CT to distinguish lipid-rich adenomas (≤ 10 HU) from LPAs (> 10 HU), obviating further investigation for the former. However, this criterion’s reliability depends critically on depends CT acquisition parameters, as tube voltage (kVp) variations substantially alter attenuation values [5]. While LPAs typically present as solid masses [6], ACs may mimic solid tumours due to cystic wall calcifications and exhibit variable internal architecture, complicating differentiation from LPAs. Enhancement characteristics further impede distinction: LPAs and some ACs show minimal enhancement, contrasting with the heterogeneous or absent enhancement typical of AGNs [7–9]. Emerging techniques such as dual-energy CT and radiomics offer potential for characterisation beyond conventional metrics, though clinical implementation requires validation [10].

Although LPAs, ACs, and AGNs are generally benign with favourable prognoses under appropriate management, preoperative differentiation carries significant clinical implications. Misdiagnosis may lead to inappropriate interventions: Functional LPAs require timely resection to prevent endocrine complications (e.g., hypertension, hypokalemia) [11–13]; Small ACs may undergo unnecessary excision instead of observation [14]; Symptomatic AGNs risk delayed surgery despite compressive symptoms (e.g., pain, organ displacement) [4, 15–18].

Given limited comparative studies of these tumours, reliable differentiation remains clinically imperative. This investigation therefore aims to identify discernible differences between LPAs, ACs, and AGNs through quantitative analysis of enhancement disparities on contrast-enhanced CT.

## Materials and methods

### Patients

**Fig. 1:**
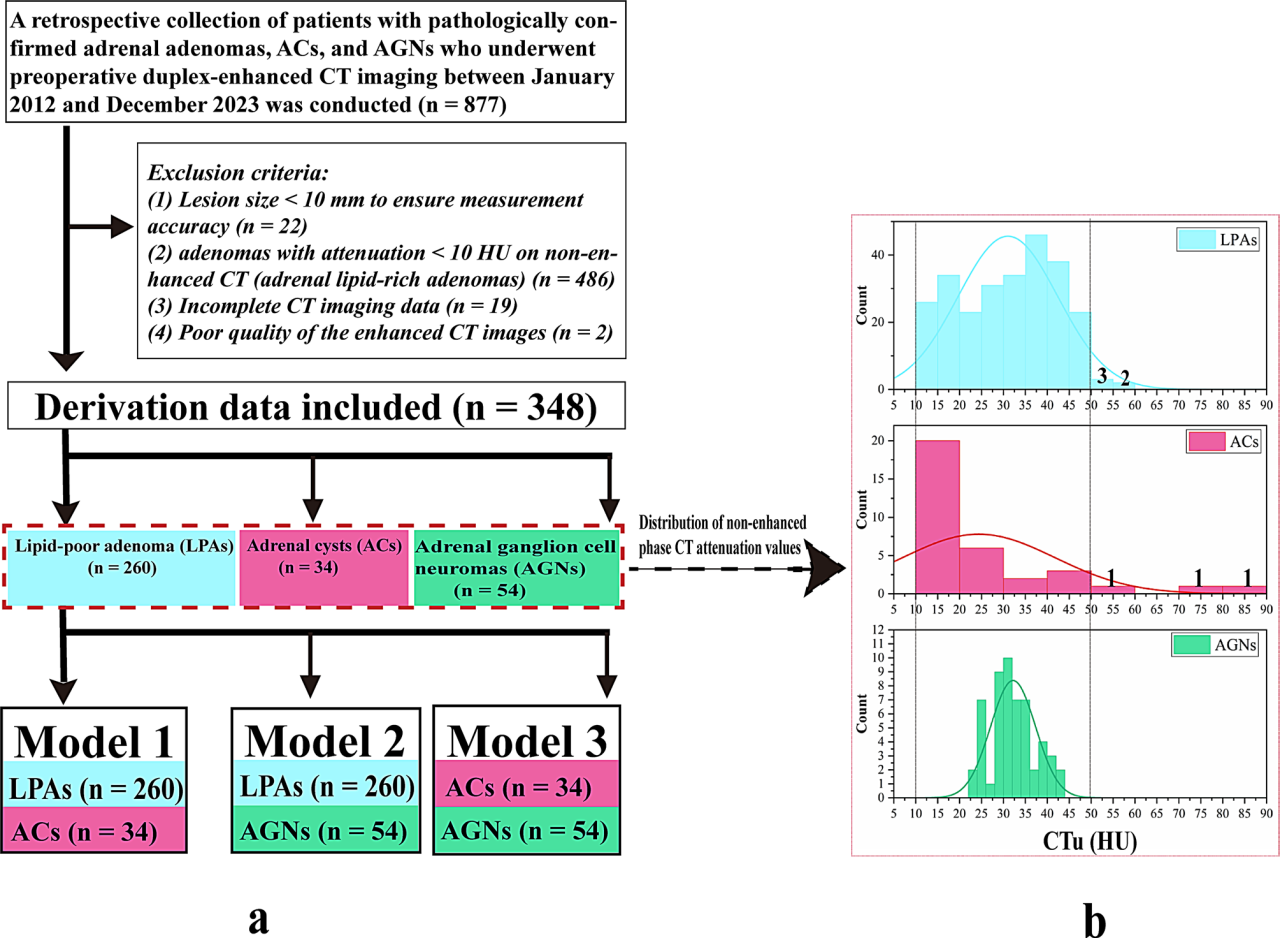
**a**. The flowchart depicts the process of patient selection and categorization. Model 1 (LPAs and ACs), Model 2 (LPAs and AGNs), and Model 3 (ACs and AGNs). **b**. CTu (HU) histograms of LPAs, ACs, and ANGs. The values on the histogram represent the frequencies of LPAs and ACs in the corresponding CTu (HU) intervals. CTu: the CT attenuation value of the unenhanced phase

This study acquired the approval of Institutional Ethics Review Board of Tongde Hospital of Zhejiang Province (approval No. 2022-183-JY), with informed consent waived because of the retrospective nature of the study. The pathological database was searched via keywords such as “adrenal lipoid adenoma,” “adrenal cyst,” and “adrenal ganglioneuroma,” and the clinical data of the patients were collected. From January 2012 to December 2023, a total of 877 adrenal adenomas, ACs, and AGNs that were pathologically confirmed and had preoperative biphasic enhanced CT images were identified. Figure 1a elaborates on the criteria used to exclude participants from this research.

### CT examination

CT images were obtained using multidetector CT (MDCT) scanners, namely, SOMATOM Sensation 16, SOMATOM Definition Flash (Siemens Healthcare, Forchheim, Germany) and LightSpeed VCT (GE Healthcare, Milwaukee, WI). All patients underwent venous catheterization in the right forearm, and a high-pressure syringe was used to inject nonionic iodine contrast material at a rate of 2.5–3.0 ml/s. The dosage of the contrast agent was calculated based on body weight, with a range of 0.8 to 1.2 ml per kilogram. CT imaging was performed in arterial phase (25–40 s) and venous phase (55–60 s). The following parameters were used for CT scanning: tube voltage, 120 kV; tube current, 200–300 mA; slice thickness, 5 mm; and matrix size, 512 × 512. The original images of 5-mm-thick slices were reconstructed into 1.5-mm-thick slices.

### Acquisition and analysis of CT images

Two blinded radiologists (10 and 18 years of experience) independently evaluated CT images. Inter-reader agreement was excellent for both categorical variables (κ ≥ 0.80) and continuous variables (intraclass correlation coefficient ≥ 0.95). Disagreements were resolved through consensus. The determined CT image features included the following: (a) CT attenuation value of unenhanced phase (CTu), CT attenuation value of arterial phase (CTa), and CT attenuation value of venous phase (CTv). *Mean CT attenuation values (CTu*,* CTa*,* CTv) were measured by placing a circular region of interest (ROI) (> 2 cm²) within the lesion*, avoiding peripheral tissues. To measure the attenuation value, the ROI area was greater than 2 cm², and these values were measured by two radiologists and averaged for the final results; (b) Aboratory indicators: normal or abnormal; (c) absolute precipitation rate (APW) = (CTa - CTv) × 100/(CTa - CTu). Relative precipitation rate (RPW) = (CTa - CTv) × 100/CTa [19]; (d) Cystic degeneration: patchy low-density lesions with no enhancement on CTu; (e) Degree of enhancement in the arterial/venous phase (DEa/vp): (CTa/v - CTu) HU; (f) Peak enhancement (DPmax): defined as the maximum value between DEap and DEvp (in HU). EPmax = DEmax/CTu; (f) GVap/GVvp: the corresponding grade variables/variables in the arterial phase; (g) Hemorrhage: CT attenuation between 55 and 90 HU, without enhancement. (h) Intratumoral vessels (observed on arterial phase images); (i) Dimensions: long diameter (LD), short diameter (SD), and the ratio of long diameter to short diameter (LD/SD); (j) Lesion location: left or right side; (k) Progressive enhancement (PE): (CTv - CTa) HU > 0 HU; (l) Symptom: presence or absence of symptoms; (m) Shape: round (LD/SD ratio ≤ 1.2), oval (LD/SD ratio > 1.2), or irregular shape.

Enhancement amplitudes (DEap, DEvp) were categorized into seven grades (0–6), generating graded variables (GVap, GVvp). For more information, refer to Table S1.

### Statistical analysis

IBM SPSS version 26.0 software was used to conduct the statistical analysis. A significance level of *p* < *0.05* was considered statistically significant. Univariate analysis was used to identify crucial clinical and CT features that distinguished LPAs, ACs, and AGNs. Quantitative variables were assessed for normality, with Student’s t test used for normally distributed variables and the Mann‒Whitney U test used for nonnormally distributed variables. Categorical variables were analyzed via the chi-square test or Fisher’s exact test. The CT features with *p* < 0.05 in the univariate analysis within each group were subjected to multivariate binary logistic regression and correlation analysis between individual factors. ROC curve analysis was performed to calculate the AUC, along with its corresponding 95% confidence interval (CI), as well as sensitivity, specificity, accuracy (ACC) measures, and the Youden index. The ACC value was computed to obtain an optimal index for differential diagnosis among these three tumors, followed by the generation of a calibration curve for validation via the HL goodness-of-fit test.

## Results

### Patient characteristics

**Fig. 2:**
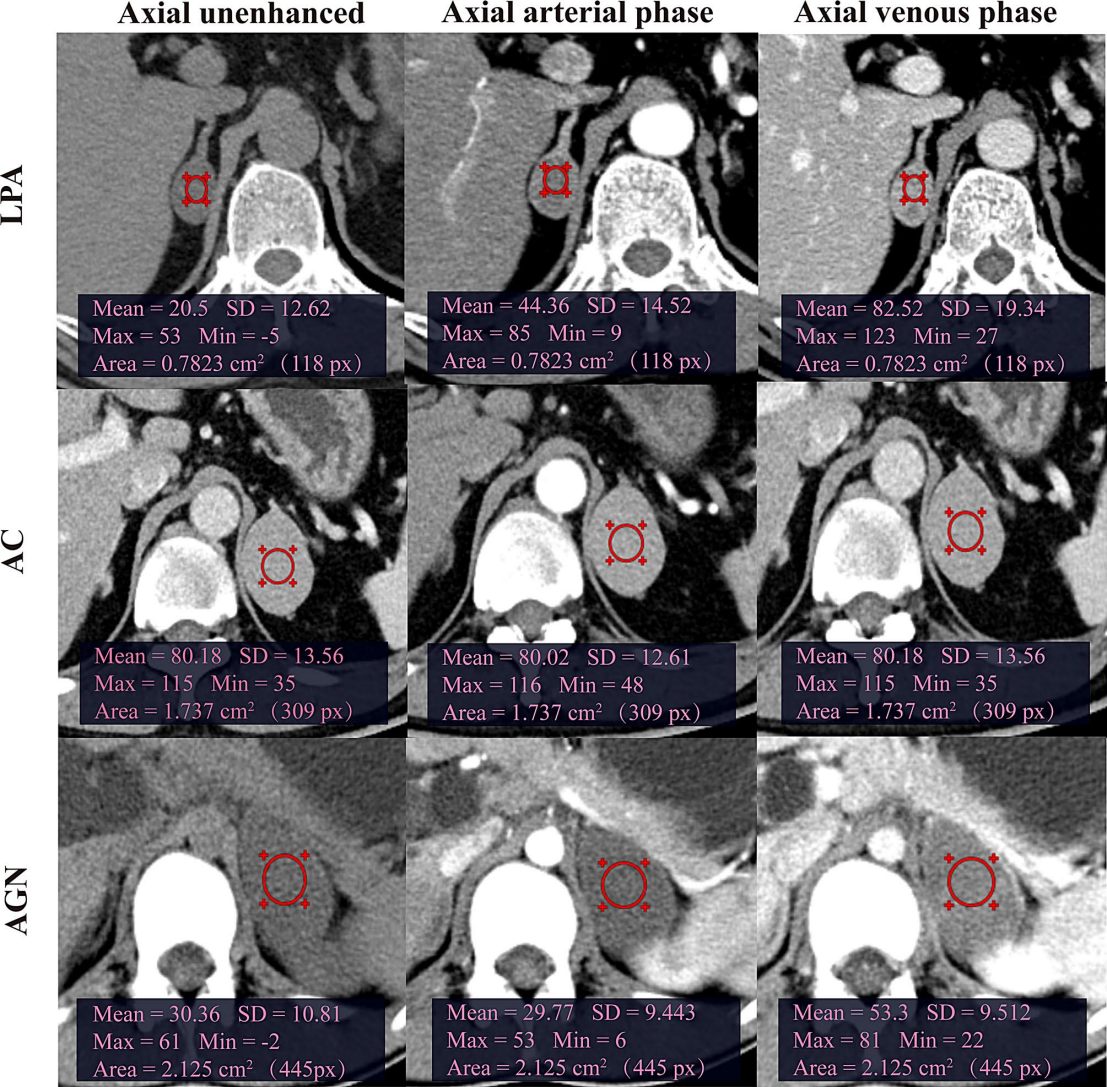
(1) The patient with a right LPA, and the CTu, CTa, and CTv values were 20.50 HU, 44.36 HU, and 82.52 HU, respectively. (2) The left AC patient with CTu, CTa, and CTv values of 80.18 HU, 80.02 HU, and 80.18 HU, respectively. (3) The patient with left AGN, and the CTu, CTa, and CTv values were 30.36 HU, 29.77 HU, and 53.30 HU, respectively. CTu = CT attenuation value of unenhanced phase. CTa = CT attenuation value of arterial phase. CTv = CT attenuation value of venous phase. AC = adrenal cyst, LPAs = lipid-poor adenoma, AGN = adrenal ganglioneuroma

Significant age differences were observed among Models 1–3 (*p < 0.05*, Table 1), whereas no significant differences in sex distribution were found across the three groups (*p > 0.05*, Table 2). Patients with only LPAs exhibited a normal distribution of ages, with a median age of 53.00 years (upper and lower quartiles: 43.00 and 59.00); ACs had an average age of 44.85 ± 2.30 years, whereas AGNs had an average age of 39.30 ± 1.85 years. The images of the clinical examples are shown in Fig. 2.

**Table 1:** Demographic and quantitative CT characteristics for pairwise comparisons of lpas, ACs and AGNs

	LPAs (*n*=260)	ACs (*n*=34)	AGNs (*n*=54)	Model _1_		Model _2_		Model _3_	
				LPAs (*n*=260)	ACs (*n*=34)	LPAs (*n*=260)	AGNs (*n*=54)	ACs (*n*=34)	AGNs (*n*=54)
				*P*_1_-value		*P*_2_-value		*P*_3_-value	
Age	53.00 (43.00, 59.00)	44.85±2.30	39.30±1.85	**0.012†**		< 0.001*		< 0.001*	
CTu (HU)	32.20(21.15,40.35)	18.40(12.0,26.70)	31.85(29.30,35.40)	**< 0.001†**		0.796†		**< 0.001†**	
CTa (HU)	68.25(52.50,93.40)	18.65(13.70,28.60)	36.25(31.40.41.00)	**< 0.001†**		**< 0.001†**		**< 0.001†**	
CTv (HU)	83.85(71.05,400.80)	19.40(14.10,30.00)	41.85(36.30,50.90)	**< 0.001†**		**< 0.001†**		**< 0.001†**	
DEap (HU)	37.05(24.95,60.80)	0.65(-0.80,2.30)	4.10(0.60,7.00)	**< 0.001†**		**< 0.001†**		**< 0.001†**	
DEvp (HU)	54.45(41.65,70.05)	0.80(-0.80,2.60)	10.15(4.80,16.40)	**< 0.001†**		**< 0.001†**		**< 0.001†**	
DEmax (HU)	58.45(44.75,74.10)	1.65(-0.30,2.80)	10.15(4.80,16.40)	**< 0.001†**		**< 0.001†**		**< 0.001†**	
EPmax	2.10(1.40,3.10)	0.10(0.00,0.10)	0.30(10.20,0.60)	**< 0.001†**		**< 0.001†**		**< 0.001†**	
APW (%)	-36.20(-101.65,2.30)	53.55(-35.50,157.10)	-115.25(-244.70,-23.60)	**< 0.001†**		**0.004†**		**< 0.001†**	
RPW (%)	-19.55(-47.90,1.75)	-1.20(-5.00,4.60)	-17.00(-28.90,-6.50)	**< 0.001†**		0.736†		**< 0.001†**	
LD (mm)	25.00(18.00,36.00)	40.50(26.00,54.00)	35.50(26.00,53.00)	**< 0.001†**		**< 0.001†**		0.589†	
SD (mm)	20.00(15.00,30.00)	29.00(22.00,42.00)	26.00(18.00,38.00)	**< 0.001†**		**< 0.001†**		0.495†	
LD/SD	1.21(1.10,1.35)	1.29(1.13,1.44)	1.37(1.20,1.50)	0.072†		**< 0.001†**		0.240†	

Note.—^*^Data are means ± standard deviation, and the statistical values are the independent sample t-test results.^†^Data do not conform to normal distribution, the median are outside parentheses, the lower quartile and the upper quartile are in parentheses, and the statistical values are Mann-Whitney U test results. P values written in bold indicate a significant difference between lesions. LPAs: lipid-poor adenomas. ACs: adrenal cysts. AGNs: adrenal ganglioneuromas. CTu/CTa/CTv = the CT attenuation value of unenhanced phase/arterial phase/venous phase; DEap = CTa - CTu; DEvp = CTv - CTu; EPmax = DEmax/CTu; DEmax is the peak value between DEap and DEvp. LD = the long diameter; SD = the short diameter; APW = absolute percentage washou, RPW = relative percentage washout

**Table 2:** Qualitative CT features for pairwise comparisons of lpas, ACs and AGNs

	LPAs (*n*=260)	ACs (*n*=34)	AGNs (*n*=54)	Model _1_		Model _2_		Model _3_	
				LPAs (*n*=260)	ACs (*n*=34)	LPAs (*n*=260)	AGNs (*n*=54)	ACs (*n*=34)	AGNs (*n*=54)
				*P*_1_-value		*P*_2_-value		*P*_3_-value	
Sex									
Men Women	114(43.8) 146(56.2)	16(47.1) 18 (52.9)	29(53.7) 25(46.3)	0.723		0.186		0.544	
GVap									
0 1 2 3 4 5 6	0(-) 4(1.5) 39(15) 45(17.3) 48(18.5) 36(13.8) 88(33.8)	14(41.2) 19(55.9) 0(-) 0(-) 0(-) 0(-) 0(-)	9(16.7) 24(44.4) 21(38.9) 0(-) 0(-) 0(-) 0(-)	**< 0.001**		**< 0.001**		**< 0.001**	
GVvp									
0 1 2 3 4 5 6	0(-) 1(0.4) 5(1.9) 16(6.2) 34(34) 46(17.7) 158(60.8)	13(38.2) 21(61.8) 0(-) 0(-) 0(-) 0(-) 0(-)	4(7.4) 10(18.5) 32(59.3) 5(9.3) 2(3.7) 1(1.9) 0(-)	**< 0.001**		**< 0.001**		**< 0.001**	
PE									
Yes No	193 (74.2) 67(25.8)	20(58.5) 14(41.2)	47(87.0) 7(13.0)	0.059		**0.044**		**0.002**	
Location									
Right Left Bilateral	118(45.4) 142(54.6) 0(-)	9(26.5) 25(7.05) 0(-)	29(53.7) 25 (46.3) 0(-)	**0.036**		0.265		**0.012**	
Shape									
Round (0) Ova1 (1) Irregular (2)	97 (37.3) 133(51.2) 30(11.5)	9 (26.5) 17 (50.0) 8 (23.5)	6(11.1) 28(51.9) 20(8.6)	0.146		**< 0.001**		0.129	
Number of lesions									
Single Multiple	185(71.2) 75(28.8)	31(91.2) 3(8.8)	52(96.3) 2(3.7)	**0.013**		**< 0.001**		0.370	
Calcification									
Yes No	55(21.2) 205(78.8)	13(38.2) 21(61.8)	12(11.5) 42(77.8)	**0.026**		0.862		0.105	
Cystic degeneration									
Yes No	16(6.2) 244(93.8)	17(5.9) 32(94.1)	1(1.9) 53(98.1)	1.000		0.324		0.557	
Hemorrhage									
Yes No	19(7.3) 241(92.7)	1(2.9) 33(97.1)	1(1.9) 53(98.1)	0.556		0.235		1.000	
Intratumoral vessel									
Yes No	108(41.5) 152(58.5)	0(-) 34(100)	6 (11.1) 48(88.9)	**< 0.001**		**< 0.001**		0.114	
‘Pointed peach’ sign									
Yes No	3(1.2) 257(98.8)	3(8.8) 31(91.2)	34(54) 20(37)	**0.022**		**< 0.001**		**< 0.001**	
HBp									
Yes No	113(43.5) 147(56.5)	1(2.9) 33(97.1)	2(3.7) 52(96.3)	**< 0.001**		**< 0.001**		1.000	
Laboratory index									
Normal Abnormal	260(100) 0(-)	18(52.9) 16(47.1)	39(72.2) 15(27.8)	**< 0.001**		**< 0.001**		0.065	
Symptom									
Yes No	33(12.7) 227(87.3)	16(47.1) 18(52.9)	10(18.5) 44(81.5)	**< 0.001**		0.257		**0.004**	

Note.—Data are numbers of lesions. Data in parentheses are percentages. P values written in bold indicate a significant difference between lesions. LPAs: Adrenal adenoma s. AGNs: Adrenal ganglioneuromas. ACs: adrenal cysts. GVap: the corresponding grade variables in the arterial phase. GVvp: the corresponding grade variables in the venous phase. PE: Progressive enhancement (0 HU < CTv-CTa). HBP: high blood pressure

### Qualitative and quantitative data analysis

In the quantitative CT characteristics shown in Table 1, the CTa, CTv, DEap, DEvp, DEmax, EPmax, and APW values were significantly different among each group of models (*p < 0.05*). LD and SD were significantly different between Model 1 and Model 2. LD/SD was significantly different only in Model 2. CTu and RPW were significantly different in Model 1 and Model 3. The median CTu value of 31.85 [29.30, 35.40] (HU) was significantly greater in the AGNs than in the ACs [18.40 (12.0, 26.70)] (HU). When CTu was greater than 50 HU, the number of cases with ACs was 3 (55–85 HU), whereas 5 cases (50–60 HU) had LPAs, and 0 cases had AGNs, as shown in Fig. 1b.

Table 2 presents the qualitative CT features. The GVap and GVvp significantly differed across all the groups (*p < 0.05*). Both the GVap and GVvp values for LPAs are greater than 0; the range of GVap for ACs is [0, 2], while the range of GVvp is [0, 1]; the GVap and GVvp range values for AGNs are [0, 2] and [0, 5], respectively (Fig. 6d). The number of lesions, intratumoral vessels, ‘pointed peach’ sign, HBp, and laboratory indices in Models 1 and 2 were significantly different. Location and symptoms displayed significant differences in Model 1 and Model 3. Shape and calcification showed significant differences only in Models 1 and 2. Cystic degeneration and hemorrhage did not significantly differ between the groups.

Table 3 shows the analysis of the four indicators with better ACCs and AUCs and corresponding 95% CIs within each group of the three sets of models (the corresponding values of the remaining indicators that were not selected are shown in Table S1). All single factors were significantly different according to the corresponding ROC curve analysis (*p < 0.05*). For the specific AUC and the corresponding ACC values, please refer to the table.

**Table 3:** Comparison of diagnostic efficacy between models

Group type	Variables	AUC	95% CI	Accuracy (%)	Sensitivity (%)	Specificity (%)	*P*-value
Model_1_	DEvp (HU)	**1.000**	1.000-1.000	99.7	99.6	100	**< 0.001**
	DEap (HU)	0.996	0.991-1.000	98.3	98.1	100	**< 0.001**
	GVap	0.993	0.985-1.000	98.3	98.5	97.1	**< 0.001**
	GVvp	0.999	0.996-1.000	99.7	99.6	100	**< 0.001**
Model_2_	DEap (HU)	**0.980**	0.967-0.993	94.6	94.6	94.4	**< 0.001**
	DEvp (HU)	0.978	0.962-0.993	94.9	95.4	92.6	**< 0.001**
	GVap	0.961	0.942-0.980	86.3	83.5	100	**< 0.001**
	GVvp	0.975	0.959-0.992	92.0	91.5	94.4	**< 0.001**
Model_3_	DEap (HU)	0.734	0.632-0.837	67.0	53.7	88.2	**< 0.001**
	DEvp (HU)	**0.892**	0.821-0.962	86.4	77.8	100	**< 0.001**
	GVap	0.725	0.620-0.829	60.2	38.9	97.1	**< 0.001**
	GVvp	0.883	0.812-0.953	84.1	74.1	100	**< 0.001**

Note. — AUC: area under the curve; DEap = CTa - CTu; DEvp = CTv - CTu; GVap: the corresponding grade variables in the arterial phase; GVvp: the corresponding grade variables in the venous phase;

Model 1: LPAs (*n*=260) and ACs (*n* = 34); Model 2: LPAs (*n*=260) and AGNs (*n* = 54); Model 3: ACs (*n*=34) and AGNs (*n* = 54). LPAs: adrenal lipid-poor adenomas; ACs: adrenal cysts; AGNs: adrenal ganglioneuromas

**Fig. 3:**
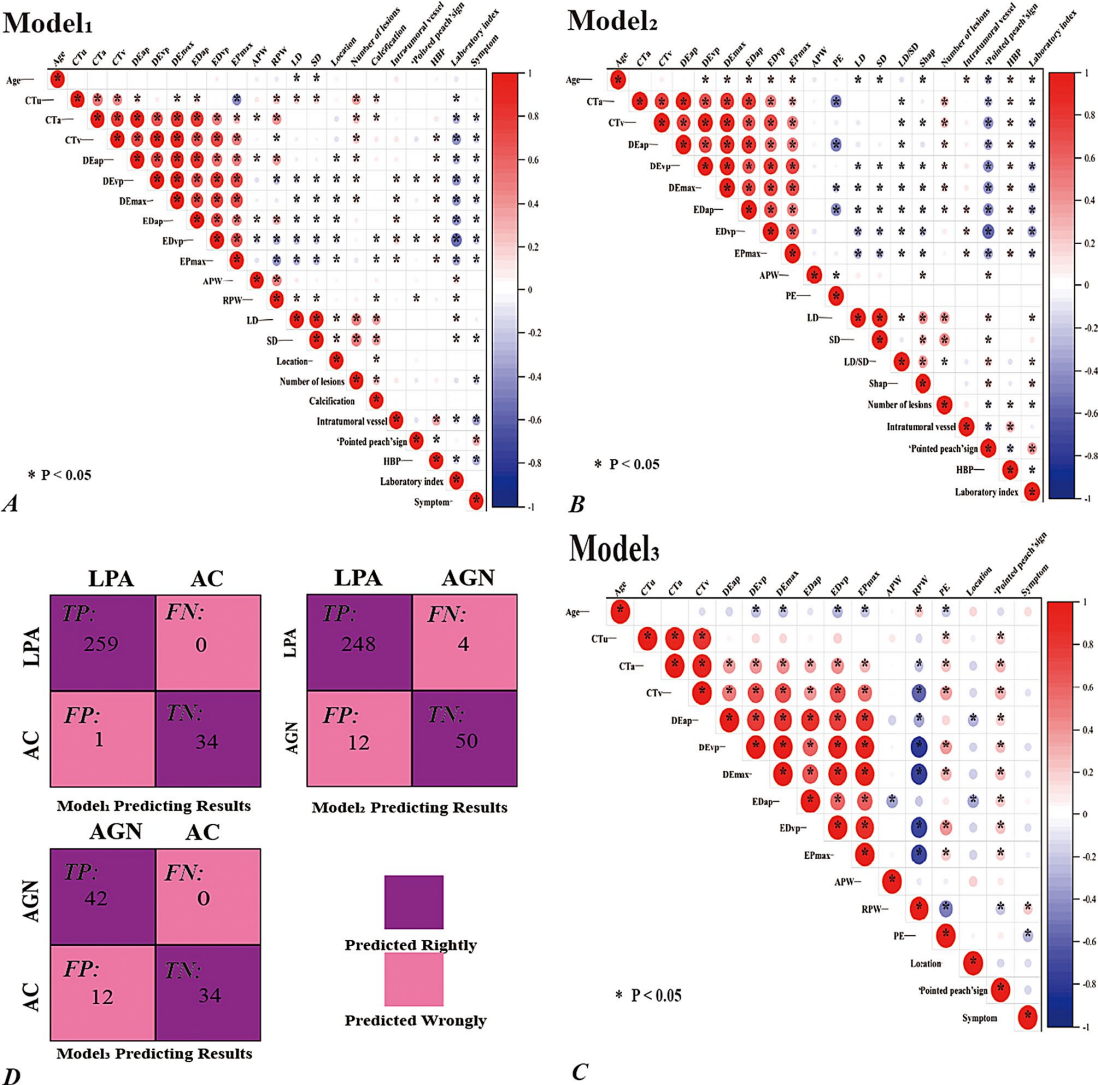
**a**-**c**. The correlation between the single factors with significant differences (*p < 0.05*) in the univariate analysis within each group of the three groups of models is shown. Red spheres indicate positive correlations, blue spheres indicate negative correlations, and larger spheres indicate stronger positive or negative correlations. The asterisk (※) indicates the threshold value of correlation significance between each single factor (*p < 0.05*), which is used as the standard to show that if there is no asterisk between each single factor, there is no positive or negative correlation; otherwise, there is correlation. Model 1: Comparison of LPAs with ACs; Model 2: Comparison of LPAs and AGNs. Model 3: Comparison of the ACs and AGNs. **d.** LPAs, ACs, and AGNs are pairs of comparative confusion matrices. Model 1: DEvp with ACs in LPAs compares the efficiency of the group. Model 2: Efficacy groups generated by DEap in the intercomparison between LPAs and AGNs. Model 3: DEvp compares the efficiency of the group of AGNs and ACs. TP: true positive; FP: false positive; FN: false negative; TN: true negative; DEap/DEvp (HU): the arterial/venous phase

Three binary logistic regression models revealed no independent risk factors (Table S2). The correlation between the single factors with significant differences (*p < 0.05*) in the univariate analysis within each of the three models is shown (Fig. 3a-c). In Model 1, out of a total of 231 pairs, 134 pairs showed a positive or significant correlation. Similarly, in Model 2, out of a total of 190 pairs, 122 pairs exhibited a positive or significant correlation, specifically involving the ‘Pointed peach’ sign with GVvp and PE, as well as CTa and DEap. Finally, Model 3 displayed a positive or significant correlation in 66 of the 120 pairs.

The confusion matrix in Fig. 3d illustrates the identification of corresponding tumors by DEvp in Model 1, DEAP in Model 2, and DEvp in Model 3. In Model 1, the correct prediction ratios for LPAs and ACs were 259/260 and 34/34, respectively. In Model 2, the correct prediction ratios for the LPAs and AGNs were 248/260 and 50/54, respectively. In Model 3, the correct prediction ratios for ACs and AGNs were 42/54 and 34/34, respectively.

**Fig. 4:**
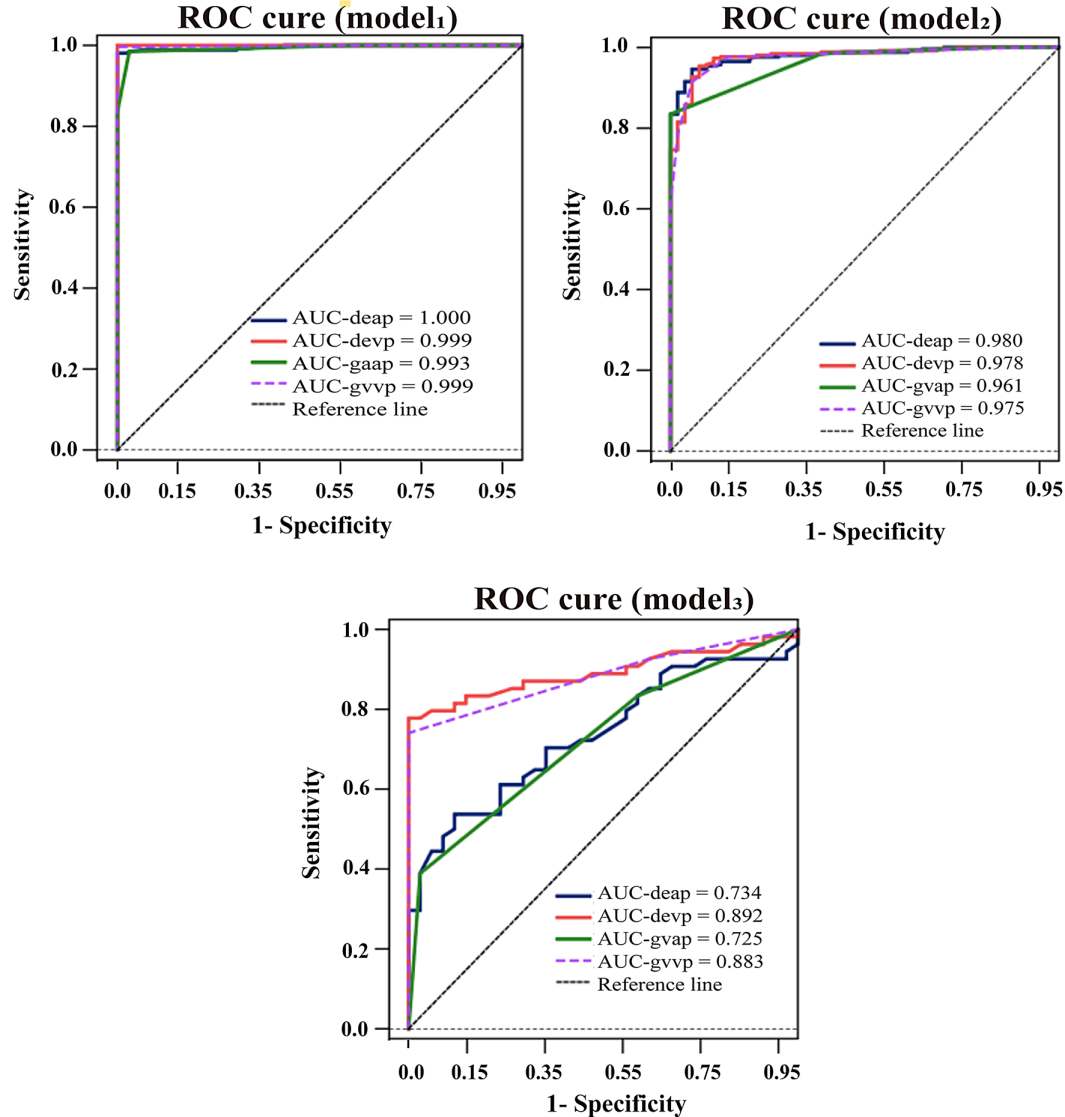
The figure shows four factors—DEap, DEvp, GVap, and GVvp—in the ROC curve for the three types of models. auc-devp: area under the curve of venous enhancement difference; auc-deap: arterial enhancement area under the curve of the difference value; auc-gvvp: area under the curve of the grade classification index of the difference value of venous enhancement. auc-gvap: arterial enhancement area under the curve of the difference value for the classification index. Model 1: Comparison between LPAs and ACs; Model 2: Comparison between LPAs and AGNs. Model 3: Comparison between ACs and AGNs. ROC: receiver operating characteristic curve; DEap/DEvp (HU): arterial/venous phase; GVap/GVvp: corresponding grade variables in the arterial/venous phase

The ROC curves of DEap, DEvp, GVap, and GVvp for the three models are presented in Fig. 4. In Model 1, the AUC values for the four single factors were 1.000, 0.999, 0.993, and 0.999; in Model 2, they were 0.980, 0.978, 0.961, and 0.975; and in Model 3, the corresponding values were 0.934, 0.892, 0.725, and 0.883.

**Fig. 5:**
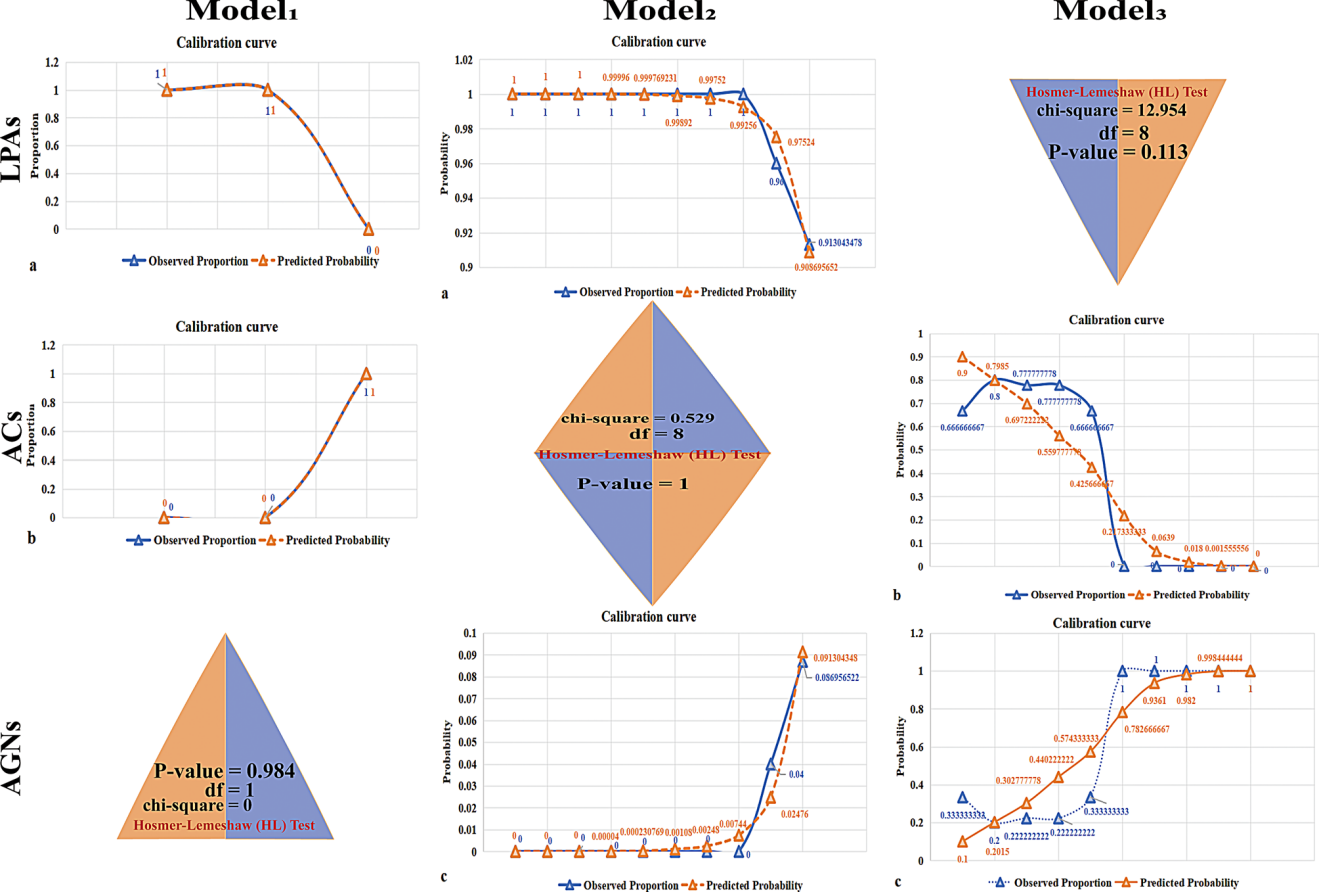
Figure shows the calibration curves of DEvp (HU) in Models 1 and 3, as well as the Hosmer-Lemeshow (HL) test values. It also includes the DEap (HU) calibration curve for Model 2 and Hosmer-Lemeshow (HL) test values. Model 1: Comparison between LPAs and ACs; Model 2: Comparison between LPAs and AGNs; Model 3: Comparison between ACs and AGN

**Fig. 6:**
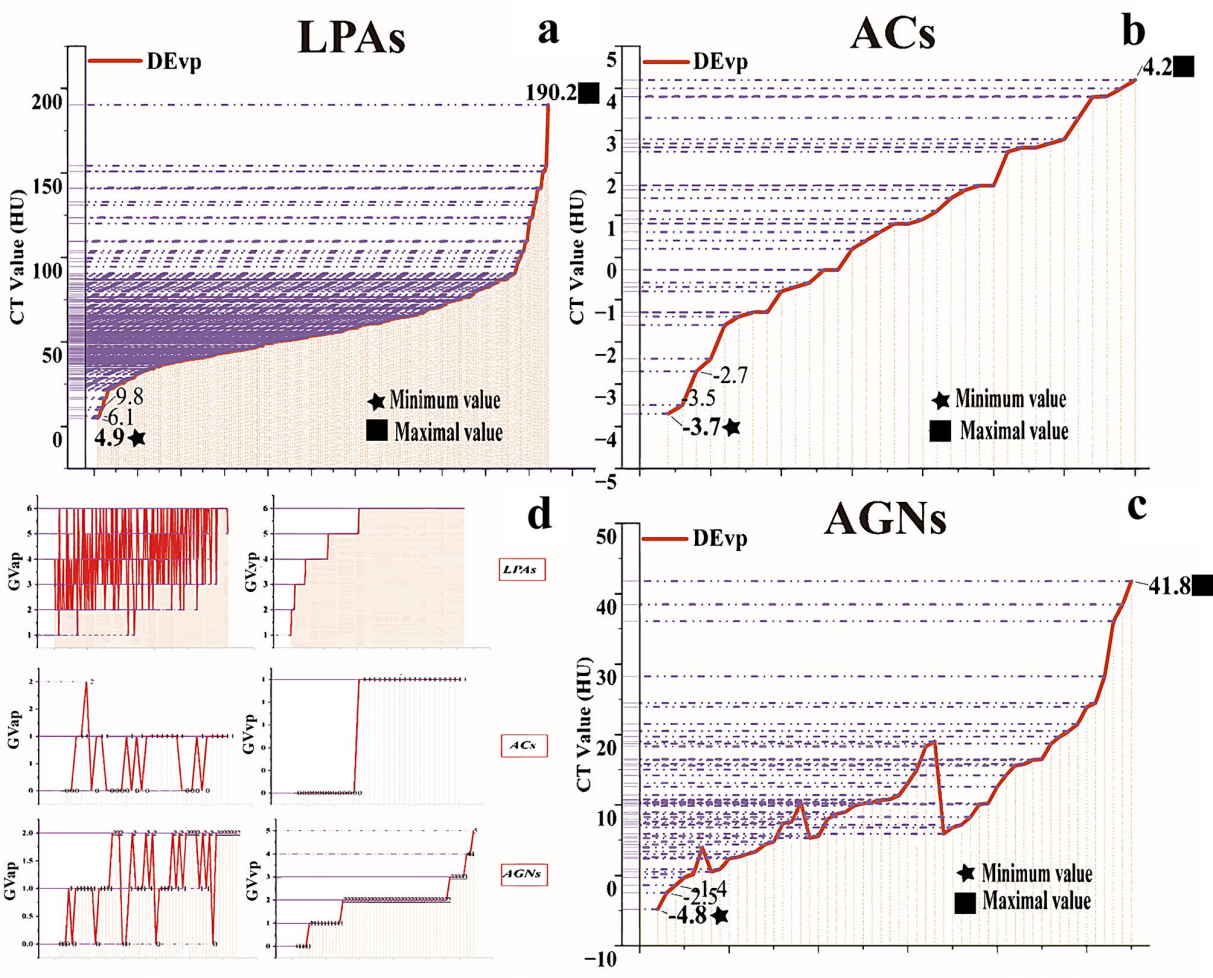
**a**-**c**. The figure shows the range of DEvp CT values (in HU) in LPA, AC, and AGN. The curve shows the change in these CT values. The minimum CT value of DEvp is represented by a pentagram, and the maximum CT value is represented by a square. **d**. This figure shows the numerical distributions of GVap and GVvp in the LPA, AC, and AGN

In the calculation of the corresponding medians and rank means for DEvp in Model 1 and Model 3 and DEap in Model 2, the P values for all three models are less than 0.05. The specific values can be found in Table 4. The P values for the goodness-of-fit tests of the three models are all greater than 0.05 (Table 5), as shown in Fig. 5. The CT value ranges for DEvp in LPAs, ACs, and AGNs are illustrated in Fig. 6a-c: [4.9, 190.2] (HU) for LPAs; [-3.7, 4.2] (HU) for ACs; and [-4.8, 41.8] (HU) for AGNs.

**Table 4:** Mann-Whitney U test between models

Model Type	Groups	Variables	Median	Rank mean	z	*P*-value
Model _1_	LPAs	DEvp (HU)	54.450	164.50	9.482	**< 0.001**
	ACs		0.800	17.50		
Model _2_	LPAs	DEap (HU)	37.050	183.43	-11.103	**< 0.001**
	AGNs		4.100	19.33		
Model _3_	ACs	DEvp (HU)	0.800	32.68	6.162	**< 0.001**
	AGNs		10.150	57.81		

Note. — LPAs: adrenal lipid-poor adenomas; ACs: adrenal cysts; AGNs: adrenal ganglioneuromas; DEap = CTa - CTu; DEvp = CTv - CTu

**Table 5:** Hosmer-Lemeshaw (HL) test

Model Type	Groups	Variables	Chi-square	DF	*P* - value
Model _1_	LPAs	DEvp	0.000	1	0.984
	ACs				
Model _2_	LPAs	DEap	0.529	8	1.000
	AGNs				
Model _3_	ACs	DEvp	12.954	8	0.113
	AGNs				

Note. — LPAs: adrenal lipid-poor adenomas; ACs: adrenal cysts; AGNs: adrenal ganglioneuromas; DEap = CTa - CTu; DEvp = CTv - CTu

## Discussion

Contrast-enhanced CT effectively differentiated LPAs, ACs, and AGNs using arterial (DEap) and venous (DEvp) phase enhancement differences. DEvp exhibited superior performance for differentiating LPAs from ACs and AGNs, while DEap optimized discrimination between LPAs and AGNs. Critically, we propose a novel criterion for non-enhancing lesions: DEvp ≤ 4.5 HU provides a biologically grounded threshold to identify non-enhancement (e.g., cysts), excluding all LPAs (minimum DEvp = 4.9 HU; Fig. 6a) while encompassing the full AC spectrum (Fig. 6b). To our knowledge, this is the first study defining such a threshold based on extreme-value analysis. Although partial overlap exists with minimally enhancing AGNs (Fig. 6c), this cutoff offers improved specificity for non-enhancing pathology. We emphasize that this threshold requires validation in larger cohorts but establishes a rational benchmark for clinical decision-making. Furthermore, our data support three exclusion principles: GVap and GVvp both equaling 0 reduces likelihood of LPA; GVap and GVvp both ≥ 2 argues against AC; and GVap ≥ 3 with GVvp = 6 diminishes probability of AGN. These parameters may aid preliminary clinical triage.

Many endocrinological societies and scholars believe that no further imaging is needed for incidental adrenal tumors with attenuation of less than 10 HU on unenhanced CT scans [6, 20], whereas LPAs have CT values greater than 10 HU on unenhanced CT in most cases. The minimum CTu value in our total sample of 348 patients was 10.1 HU for LPAs, 22.6 HU for AGNs, and 10.0 HU for ACs, all of which are greater than 10 HU, consistent with previous studies. AGNs had higher CTu values than ACs (median: 31.85 HU vs. 18.40 HU, *p* < 0.05; Table 1), consistent with Shao et al. [21]. ACs had lower CTu/CTa/CTv than LPAs or AGNs (*p < 0.05*), consistent with their near-water density, which is consistent with the fact that cysts generally have a density close to that of water [22, 23]. However, in our study, there was a significant difference between the CTu values of ACs and those of LPAs and AGNs (*p < 0.05*), but there was no significant difference between the CTu values of LPAs and AGNs (*p > 0.05*). Notably, elevated CTu (> 50 HU) occurred in both ACs and LPAs (Fig. 1b), underscoring the need for enhanced differentiation.

Most LPAs were smaller than ACs and AGNs. Supporting size-based intervention thresholds [19], ACs and AGNs exceeded 50 mm more frequently than LPAs (Table 1). This suggests that *accurate differentiation is clinically* essential between LPA, AC, and AGN and that our sample selection is reasonable. In terms of the location of incidence, a significant difference was observed between Model 1 and Model 3 (*p < 0.05*). However, no significant difference was found in Model 2 (*p = 0.265*). Additionally, all 348 patients were affected unilaterally. It is reported that adrenal cysts are predominantly unilateral (> 80%) [24]. Considering the sample size and case selection, our research supports this finding. In terms of calcification, Model 1 was significantly different (*p < 0.05*), whereas Models 2 and 3 were not significantly different (*p > 0.05)*. Calcification frequency differed significantly between ACs and other lesions (Table 2), while AGNs showed higher ‘pointed peach’ sign prevalence (54% vs. ≤8.8%; p < 0.05). In previous studies on AGNs [15, 25–28], calcification (at a rate of 30–60%) and the ‘pointed peach’ sign were important diagnostic features. Our findings concerning the ‘pointed peach’ sign are consistent with these findings, but there is a difference in calcification.

Notably, APW and RPW percentage washout represent established metrics for adrenal lesion characterization. APW demonstrated statistically significant differences across all lesion groups (*p* < 0.05, Table 1), aligning with its traditional diagnostic utility. RPW achieved significance in Models 1 and 3 (*p* < 0.001) but not Model 2 (*p* = 0.736). Nevertheless, comparative evaluation revealed that arterial (DEap) and venous phase (DEvp) enhancement differences exhibited consistently superior diagnostic performance across models. This was evidenced by their higher AUC values (e.g., DEvp AUC = 1.000 in Model 1 versus historically lower washout AUCs in adenoma/non-adenoma studies). Critically, DEvp and DEap enabled clearer discriminatory thresholds and clinical rules (e.g., DEvp ≤ 4.5 HU indicating non-enhancement; GVap/GVvp combinations excluding lesion types). Although analyzed for completenessWashout metrics (APW/RPW) were analyzed for completeness but proved inferior to enhancement differences for discriminating these lesions. Consequently, although washout metrics retain conceptual relevance, DEap and DEvp proved more potent for differentiating LPAs, ACs, and AGNs. The analysis therefore prioritized these enhancement difference parameters.

We have developed Model 1 and Model 2, which outperform existing models. DEvp achieved optimal diagnostic performance for Models 1/3 (AUC = 1.000/0.892), while DEap excelled in Model 2 (AUC = 0.980; Table 3). For all three models, the HL test p values were 0.984, 1.000, and 0.113, respectively, indicating a good fit. Most of the previous studies have focused primarily on discussing ACs and AGNs in terms of individual cases, resulting in a scarcity of comparative reports regarding diverse research methods for differential diagnosis among these three types of pathological changes. Some scholars, such as Zhang et al., have explored the differential diagnosis between LPAs and nonadenomas [29]. They successfully established an optimal model with an AUC of 0.96, a sensitivity of 92.9%, and a specificity of 88.1% for distinguishing between LPAs and adenomas.

The differential utility of DEap across models is rooted in lesion-specific enhancement kinetics. In Model 2 (LPAs vs. AGNs), DEap achieved superior diagnostic performance (AUC = 0.980) because AGNs showed minimal arterial enhancement (median ΔDEap = 4.10 HU), contrasting sharply with LPAs (37.05 HU; *p* < 0.001; Table 4), reflecting adrenocortical vascular patterns [6]. This generated a 9-fold arterial-phase ΔHU (32.95 HU), maximizing interlesion contrast. In the venous phase, AGNs’ delayed enhancement (median DEvp: 10.15 HU) partially converged with LPAs’ persistent enhancement (54.45 HU), reducing discrimination (Δ = 44.30 HU). For Model 1 (LPAs vs. ACs), venous-phase metrics dominated due to ACs’ near-absent enhancement (DEvp: 0.80 HU vs. LPAs: 54.45 HU; Δ = 53.65 HU). In Model 3 (ACs vs. AGNs), the venous phase remained optimal as AGNs’ delayed enhancement widened their separation from ACs (Δ = 9.35 HU vs. arterial Δ = 3.45 HU). Critically, our 55–60 s venous phase—an early delayed phase—limited washout characterization compared to guideline-recommended 15-min delayed imaging [1], further diminishing venous-phase utility for slowly enhancing AGNs in Model 2. Despite arterial phase not being standard for adrenal protocols [1], its inclusion significantly improved AGN vs. LPA discrimination. We recommend dual-phase CT when AGN is suspected.

Notably, Zhang et al. [29], O’Shea et al. [30], and Cao et al. [31] reported that the distribution histogram established by the HU median (or mean) has a certain correlation in their studies distinguishing LPAs from other tissues, and we agreed with them. In our research, all three groups of models showed significant differences (*p < 0.05)*, indicating that our model was reasonable and that it was feasible to distinguish the three lesions via DEvp or DEap (Table 4).

### Limitations

Several limitations warrant acknowledgment. First, the absence of an independent validation cohort restricts comprehensive performance assessment of our models, though internal validation via the Hosmer-Lemeshow test indicated good fit (*p* >0.05). Second, the sample distribution was uneven across lesion types (LPA: 260; AC: 34; AGN: 54), reflecting their relative incidences but potentially affecting generalizability. Third, retrospective collection led to incomplete biological data for some patients, possibly influencing ancillary analyses. Future multi-center studies with balanced cohorts and prospective designs should validate our enhancement thresholds (e.g., DEvp ≤ 4.5 HU for non-enhancing lesions) and explore advanced techniques like radiomics.

## Conclusions

Our analysis demonstrates that difference values of the arterial and venous phases and their corresponding grading variables on enhanced CT images can effectively aid in distinguishing these three types of adrenal lesions, providing valuable reference for clinical diagnosis. Prospective studies **are needed to** validate these findings and explore additional methods to enhance diagnostic accuracy.

## Supplementary Information

Below is the link to the electronic supplementary material.

Supplementary Material 1

## Data Availability

The sequence data that support the findings of this study have been deposited in the relevant files.

## Abbreviations

ACs: Adrenal cysts
ACC: Accuracy
AGNs: Adrenal ganglioneuromas
APW: Absolute percentage washout
AUC: Area under the curve
CI: Confidence interval
CTu: CT attenuation value of unenhanced phase
CTa: CT attenuation value of arterial phase
CTv: CT attenuation value of venous phase
DEap: The arterial phase
DEvp: The venous phase
DEmax: The peak value between DEap and DEvp
EPmax: DEmax/CTu
GVap: The corresponding grade variables in the arterial phase
GVvp: The corresponding grade variables in the venous phase
HBP: High blood pressure
Hosmer‒Lemeshow test: HL test
LD: The long diameter
LPAs: Lipid-poor adenomas
PE: Progressive enhancement
ROC: Receiver operating characteristic curves
ROI: Region of interest
RPW: Relative percentage washout
SD: The short diameter
IQR: Interquartile range
