# Supplementary material for: New heights in CT differentiation of adrenal lesions and a rational definition of non-enhancement
Source: BMC Med Imaging. 2025 Sep 26;25:374. doi: 10.1186/s12880-025-01916-6 (PMC12466030; doi:10.1186/s12880-025-01916-6)
Supplement: Supplementary file 1 — Supplementary Material 1 [file 12880_2025_1916_MOESM1_ESM.docx]

**Table S1. Exploring different segmentation methods and comparing the diagnostic efficacy among the corresponding models**

| **Explo--rations** | **Variables** | | **Mo-del** | **Area under the cure** | | | | | | **Coordinates of the cure** | | | | |
| --- | --- | --- | --- | --- | --- | --- | --- | --- | --- | --- | --- | --- | --- | --- |
|  | **GVap/GVvp** | **DEap/DEvp** |  | **Variables** | **Area** | **Std Error^＊^** | **Asymptotic Sig**b | **95% CI** | | **Cutoff value** | **Sensitivity** | **Specificity** | **1-Specificity** | **Youden indx** |
|  |  |  |  |  |  |  |  | **Low Bound** | **Upper Bound** |  |  |  |  |  |
| E1* | 0 | ≤ 0 | 1 | DEap | 0.996 | 0.003 | 0.000 | 0.991 | 1.000 | 6.3 | 0.981 | 0 | 1 | 0.981 |
|  |  |  |  | **DEvp** | **1.000** | **0.000** | **0.000** | **1.000** | **1.000** | **5.5** | **0.996** | **0** | **1** | **0.996** |
|  | 1 | 0 **<** CT value ≤ 5 |  | GVap | 0.993 | 0.004 | 0.000 | 0.985 | 1.000 | 1.5 | 0.985 | 0.029 | 0.971 | 0.956 |
|  |  |  |  | GVvp | 0.999 | 0.001 | 0.000 | 0.996 | 1.000 | 1.5 | 0.996 | 0 | 1 | 0.956 |
|  | 2 | 5 **<** CT value ≤ 20 | 2 | **DEap** | **0.980** | **0.007** | **0.000** | **0.967** | **0.993** | **11.3** | **0.946** | **0.056** | **0.944** | **0.890** |
|  |  |  |  | DEvp | 0.978 | 0.008 | 0.000 | 0.962 | 0.993 | 24.7 | 0.954 | 0.074 | 0.926 | 0.880 |
|  | 3 | 20 **<** CT value ≤ 30 |  | GVap | 0.961 | 0.010 | 0.000 | 0.942 | 0.980 | 2.5 | 0.835 | 0 | 1 | 0.835 |
|  |  |  |  | GVvp | 0.975 | 0.009 | 0.000 | 0.959 | 0.992 | 3.5 | 0.915 | 0.056 | 0.944 | 0.859 |
|  | 4 | 30 **<** CT value ≤ 40 | 3 | DEap | 0.734 | 0.052 | 0.000 | 0.632 | 0.837 | 3.75 | 0.537 | 0.118 | 0.882 | 0.419 |
|  |  |  |  | **DEvp** | **0.892** | **0.036** | **0.000** | **0.821** | **0.962** | **4.3** | **0.778** | **0** | **1** | **0.778** |
|  | 5 | 40 **<** CT value ≤ 50 |  | GVap | 0.725 | 0.053 | 0.000 | 0.620 | 0.829 | 1.5 | 0.389 | 0.029 | 0.971 | 0.360 |
|  | 6 | CT value > 50 |  | GVvp | 0.883 | 0.036 | 0.000 | 0.812 | 0.953 | 1.5 | 0.741 | 0 | 1 | 0.741 |
| E2 | 0 | ≤ 0 | 1 | DEap | 0.996 | 0.003 | 0.000 | 0.991 | 1.000 | 6.3 | 0.981 | 0 | 1 | 0.981 |
|  |  |  |  | DEvp | 1.000 | 0.000 | 0.000 | 1.000 | 1.000 | 5.5 | 0.996 | 0 | 1 | 0.996 |
|  | 1 | 0 **<** CT value ≤ 5 |  | GVap | 0.994 | 0.003 | 0.000 | 0.985 | 1.000 | 1.5 | 0.985 | 0.029 | 0.971 | 0.956 |
|  |  |  |  | GVvp | 0.999 | 0.001 | 0.000 | 0.996 | 1.000 | 1.5 | 0.996 | 0 | 1 | ***0.996*** |
|  | 2 | 5 **<** CT value ≤ 15 | 2 | DEap | 0.980 | 0.007 | 0.000 | 0.967 | 0.993 | 11.3 | 0.946 | 0.056 | 0.944 | 0.890 |
|  |  |  |  | DEvp | 0.978 | 0.008 | 0.000 | 0.962 | 0.993 | 24.7 | 0.954 | 0.074 | 0.926 | 0.880 |
|  | 3 | 15 **<** CT value ≤ 25 |  | GVap | ***0.971*** | ***0.008*** | 0.000 | ***0.954*** | ***0.987*** | 2.5 | ***0.904*** | ***0.037*** | ***0.963*** | ***0.867*** |
|  |  |  |  | GVvp | ***0.972*** | ***0.010*** | 0.000 | ***0.953*** | ***0.991*** | 3.5 | ***0.950*** | ***0.074*** | ***0.926*** | ***0.876*** |
|  | 4 | 25 **<** CT value ≤ 35 | 3 | DEap | 0.734 | 0.052 | 0.000 | 0.632 | 0.837 | 3.75 | 0.537 | 0.118 | 0.882 | 0.419 |
|  |  |  |  | DEvp | 0.892 | 0.036 | 0.000 | 0.821 | 0.962 | 4.3 | 0.778 | 0 | 1 | 0.778 |
|  | 5 | 35 **<** CT value ≤ 50 |  | GVap | 0.725 | 0.053 | 0.000 | 0.621 | 0.829 | 2.5 | ***0.037*** | ***0*** | ***1*** | ***0.037*** |
|  | 6 | CT value > 50 |  | GVvp | 0.883 | 0.036 | 0.000 | 0.812 | 0.953 | 1.5 | 0.741 | 0 | 1 | 0.741 |
| E3 | 0 | ≤ 0 | 1 | DEap | 0.996 | 0.003 | 0.000 | 0.991 | 1.000 | 6.3 | 0.981 | 0 | 1 | 0.981 |
|  |  |  |  | DEvp | 1.000 | 0.000 | 0.000 | 1.000 | 1.000 | 5.5 | 0.996 | 0 | 1 | 0.996 |
|  | 1 | 0 **<** CT value ≤ 10 |  | GVap | ***0.992*** | ***0.005*** | 0.000 | ***0.982*** | 1.000 | ***0.5*** | 0.985 | 0.029 | 0.971 | 0.956 |
|  |  |  |  | GVvp | ***0.998*** | ***0.002*** | 0.000 | ***0.994*** | 1.000 | ***0.5*** | 0.996 | 0 | 1 | ***0.996*** |
|  | 2 | 10 **<** CT value ≤ 20 | 2 | DEap | 0.980 | 0.007 | 0.000 | 0.967 | 0.993 | 11.3 | 0.946 | 0.056 | 0.944 | 0.890 |
|  |  |  |  | DEvp | 0.978 | 0.008 | 0.000 | 0.962 | 0.993 | 24.7 | 0.954 | 0.074 | 0.926 | 0.880 |
|  | 3 | 20 **<** CT value ≤ 30 |  | GVap | ***0.975*** | ***0.008*** | 0.000 | ***0.960*** | ***0.990*** | ***1.5*** | ***0.946*** | ***0.093*** | ***0.907*** | ***0.853*** |
|  |  |  |  | GVvp | 0.975 | ***0.009*** | 0.000 | 0.959 | 0.992 | 3.5 | 0.915 | 0.056 | 0.944 | 0.859 |
|  | 4 | 30 **<** CT value ≤ 45 | 3 | DEap | 0.734 | 0.052 | 0.000 | 0.632 | 0.837 | 3.75 | 0.537 | 0.118 | 0.882 | 0.419 |
|  |  |  |  | DEvp | 0.892 | 0.036 | 0.000 | 0.821 | 0.962 | 4.3 | 0.778 | 0 | 1 | 0.778 |
|  | 5 | CT value > 45 |  | GVap | ***0.681*** | ***0.056*** | ***0.004*** | ***0.572*** | ***0.790*** | ***0.5*** | 0.389 | 0.029 | 0.971 | 0.360 |
|  |  |  |  | GVvp | ***0.870*** | ***0.038*** | 0.000 | ***0.795*** | ***0.946*** | ***0.5*** | 0.741 | 0 | 1 | 0.741 |
| E4 | 0 | ≤ 7.5 | 1 | DEap | 0.996 | 0.003 | 0.000 | 0.991 | 1.000 | 6.3 | 0.981 | 0 | 1 | 0.981 |
|  |  |  |  | DEvp | 1.000 | 0.000 | 0.000 | 1.000 | 1.000 | 5.5 | 0.996 | 0 | 1 | 0.996 |
|  | 1 | 7.5 **<** CT value ≤ 12.5 |  | GVap | ***0.985*** | ***0.006*** | 0.000 | ***0.972*** | ***0.997*** | ***0.5*** | ***0.969*** | ***0*** | ***1*** | ***0.969*** |
|  |  |  |  | GVvp | ***0.996*** | ***0.003*** | 0.000 | ***0.990*** | 1.000 | ***0.5*** | ***0.992*** | 0 | 1 | 0.992 |
|  | 2 | 12.5 **<** CT value ≤ 22.5 | 2 | DEap | 0.980 | 0.007 | 0.000 | ***0.967*** | 0.993 | 11.3 | 0.946 | 0.056 | 0.944 | 0.890 |
|  |  |  |  | DEvp | 0.978 | 0.008 | 0.000 | 0.962 | 0.993 | 24.7 | 0.954 | 0.074 | 0.926 | 0.880 |
|  | 3 | 22.5 **<** CT value ≤ 35 |  | GVap | ***0.973*** | ***0.008*** | 0.000 | ***0.956*** | ***0.989*** | ***1.5*** | ***0.931*** | ***0.056*** | ***0.944*** | ***0.875*** |
|  |  |  |  | GVvp | ***0.971*** | ***0.010*** | 0.000 | ***0.952*** | **0.990** | ***2.5*** | ***0.965*** | ***0.111*** | ***0.889*** | 0.854 |
|  | 4 | 35 **<** CT value ≤ 47.5 | 3 | DEap | 0.734 | 0.052 | 0.000 | 0.632 | 0.837 | 3.75 | 0.537 | 0.118 | 0.882 | 0.419 |
|  |  |  |  | DEvp | 0.892 | 0.036 | 0.000 | 0.821 | 0.962 | 4.3 | 0.778 | 0 | 1 | 0.778 |
|  | 5 | CT value > 47.5 |  | GVap | ***0.602*** | ***0.060*** | ***0.109*** | ***0.485*** | ***0.719*** | ***0.5*** | ***0.204*** | ***0*** | 1 | ***0.204*** |
|  |  |  |  | GVvp | ***0.815*** | ***0.045*** | 0.000 | ***0.727*** | ***0.903*** | ***0.5*** | ***0.630*** | 0 | 1 | ***0.630*** |
| E5 | 0 | ≤ 0 | 1 | DEap | 0.996 | 0.003 | 0.000 | 0.991 | 1.000 | 6.3 | 0.981 | 0 | 1 | 0.981 |
|  |  |  |  | DEvp | 1.000 | 0.000 | 0.000 | 1.000 | 1.000 | 5.5 | 0.996 | 0 | 1 | 0.996 |
|  | 1 | 0 **<** CT value ≤ 5 |  | GVap | ***0.990*** | ***0.006*** | 0.000 | ***0.978*** | 1.000 | 1.5 | 0.985 | 0.029 | 0.971 | 0.956 |
|  |  |  |  | GVvp | 0.999 | 0.001 | 0.000 | 0.996 | 1.000 | 1.5 | 0.996 | 0 | 1 | 0.996 |
|  | 2 | 5 **<** CT value ≤ 30 | 2 | DEap | 0.980 | 0.007 | 0.000 | 0.967 | 0.993 | 11.3 | 0.946 | 0.056 | 0.944 | 0.890 |
|  |  |  |  | DEvp | 0.978 | 0.008 | 0.000 | 0.962 | 0.993 | ***21.8*** | ***0.973*** | ***0.111*** | ***0.889*** | ***0.862*** |
|  | 3 | 30 **<** CT value ≤ 50 |  | GVap | ***0.928*** | ***0.016*** | 0.000 | ***0.896*** | ***0.959*** | 2.5 | ***0.662*** | 0 | 1 | ***0.662*** |
|  |  |  |  | GVvp | ***0.956*** | ***0.110*** | 0.000 | ***0.934*** | ***0.978*** | ***2.5*** | 0.915 | 0.056 | 0.944 | 0.859 |
|  | 4 | CT value > 50 | 3 | DEap | 0.734 | 0.052 | 0.000 | 0.632 | 0.837 | 3.75 | 0.537 | 0.118 | 0.882 | 0.419 |
|  |  |  |  | DEvp | 0.892 | 0.036 | 0.000 | 0.821 | 0.962 | 4.3 | 0.778 | 0 | 1 | 0.778 |
|  |  |  |  | GVap | 0.725 | 0.053 | 0.000 | 0.620 | 0.829 | 1.5 | 0.389 | 0.029 | 0.971 | 0.360 |
|  |  |  |  | GVvp | 0.883 | 0.036 | 0.000 | 0.812 | 0.953 | 1.5 | 0.741 | 0 | 1 | 0.741 |

Note.—*****The groupings used in this study. Italicized text produces different results from the subgroups selected for this study. Bold text indicates the factors that produced optimal performance within the selected groups in this study. DEap: the degree of enhancement in the arterial phase; DEvp: the degree of enhancement in the venous phase; GVap: the corresponding grade variables in the arterial phase; GVvp: the corresponding grade variables in the venous phase. LPAs: Adrenal adenoma s. AGNs: Adrenal ganglioneuromas. ACs: adrenal cysts. Model 1: LPAs (n = 260) and ACs ((n = 34); Model 2: LPAs (n = 260) and AGNs ((n = 54); Model 3: ACs (n = 34) and AGNs (n = 54).

**Table S2. Binary logistic regression results for three group models (p-value)**

| **Variables** | **Model _1_** | | **Model _2_** | | **Model _3_** | |
| --- | --- | --- | --- | --- | --- | --- |
|  | LPAs  (n=260) | ACs  (n=34 | LPAs  (n=260) | AGNs  (n=54) | ACs  (n=34) | AGNs  (n=54) |
|  | *P_1_-value* | | *P_2_-value* | | *P_3_-value* | |
| Age | 1.000 | | 0.996 | | 0.998 | |
| CTU | 0.999 | | **×** | | 0.996 | |
| CTA | 1.000 | | 0.993 | | 0.987 | |
| CTV | 0.999 | | 0.994 | | 0.960 | |
| DEmax | 0.999 | | 0.997 | | 0.961 | |
| EPmax | 0.999 | | 0.998 | | 0.968 | |
| APW | 1.000 | | 1.000 | | 0.955 | |
| RPW | 1.000 | | **×** | | 0.965 | |
| LD | 0.999 | | 0.999 | | **×** | |
| SD | 0.999 | | 0.999 | | **×** | |
| LD/SD | **×** | | 0.999 | | **×** | |
| Sex | **×** | | **×** | | **×** | |
| GVap | 0.999 | | 0.997 | | 0.987 | |
| GVvp | 0.998 | | 0.999 | | 0.955 | |
| PE | **×** | | 0.998 | | 0.956 | |
| Location | 0.999 | | **×** | | 0.954 | |
| shape | **×** | | 0.999 | | **×** | |
| Number of lesions） | 0.999 | | 0.999 | | **×** | |
| Calcification | 0.999 | | **×** | | **×** | |
| Cystic degeneration | **×** | | **×** | | **×** | |
| Hemorrhage | **×** | | **×** | | **×** | |
| Intratumoral vessel | 0.999 | | 0.998 | | **×** | |
| ‘Pointed peach’ sign | 1.000 | | 0.997 | | 0.956 | |
| HBP | 1.000 | | 0.995 | | **×** | |
| Laboratory index | 0.999 | | 0.998 | | **×** | |
| Symptom | 0.999 | | **×** | | 0.958 | |

Cross sign (×): This single factor showed no significant difference in the univariate analysis (*p ≥ 0.05*). LPAs: Adrenal adenoma s. AGNs: Adrenal ganglioneuromas. ACs: adrenal cysts. CTu/CTa/CTv = the CT attenuation value of unenhanced phase/arterial phase/venous phase; EPmax = DEmax/CTu; DEmax is the [peak](javascript:void(0);) value between DEap and DEvp; LD = the long diameter; SD = the short diameter; APW = absolute percentage washou; RPW = relative percentage washout; PE: Progressive enhancement (0 HU < CTv-CTa); HBP: high blood pressure.
